# Genomic analysis of the chromosome 15q11-q13 Prader-Willi syndrome region and characterization of transcripts for GOLGA8E and WHCD1L1 from the proximal breakpoint region

**DOI:** 10.1186/1471-2164-9-50

**Published:** 2008-01-28

**Authors:** Yong-hui Jiang, Kekio Wauki, Qian Liu, Jan Bressler, Yanzhen Pan, Catherine D Kashork, Lisa G Shaffer, Arthur L Beaudet

**Affiliations:** 1 Departments of Molecular and Human Genetics, Baylor College of Medicine, Houston, TX 77030, USA; 2 Signature Genomics Laboratories, LLC, 120 North Pine Street, Suite 242C, Spokane, WA 99202, USA; 3 Shinshu University School of Medicine, Dept of Medical Genetics, 3-1-1 Asahi, Nagano, Matsumoto 390-8621, Japan

## Abstract

**Background:**

Prader-Willi syndrome (PWS) is a neurobehavioral disorder characterized by neonatal hypotonia, childhood obesity, dysmorphic features, hypogonadism, mental retardation, and behavioral problems. Although PWS is most often caused by a paternal interstitial deletion of a 6-Mb region of chromosome 15q11-q13, the identity of the exact protein coding or noncoding RNAs whose deficiency produces the PWS phenotype is uncertain. There are also reports describing a PWS-like phenotype in a subset of patients with full mutations in the *FMR1 *(fragile X mental retardation 1) gene. Taking advantage of the human genome sequence, we have performed extensive sequence analysis and molecular studies for the PWS candidate region.

**Results:**

We have characterized transcripts for the first time for two UCSC Genome Browser predicted protein-coding genes, *GOLGA8E *(golgin subfamily a, 8E) and *WHDC1L1 *(WAS protein homology region containing 1-like 1) and have further characterized two previously reported genes, *CYF1P1 *and *NIPA2*; all four genes are in the region close to the proximal/centromeric deletion breakpoint (BP1). *GOLGA8E* belongs to the golgin subfamily of coiled-coil proteins associated with the Golgi apparatus. Six out of 16 golgin subfamily proteins in the human genome have been mapped in the chromosome 15q11-q13 and 15q24-q26 regions. We have also identified more than 38 copies of *GOLGA8E*-like sequence in the 15q11-q14 and 15q23-q26 regions which supports the presence of a *GOLGA8E*-associated low copy repeat (LCR). Analysis of the 15q11-q13 region by PFGE also revealed a polymorphic region between BP1 and BP2. *WHDC1L1 *is a novel gene with similarity to mouse *Whdc1 *(WAS protein homology region 2 domain containing 1) and human JMY protein (junction-mediating and regulatory protein). Expression analysis of cultured human cells and brain tissues from PWS patients indicates that *CYFIP1 *and *NIPA2* are biallelically expressed. However, we were not able to determine the allele-specific expression pattern for *GOLGA8E *and *WHDC1L1 *because these two genes have highly related sequences that might also be expressed.

**Conclusion:**

We have presented an updated version of a sequence-based physical map for a complex chromosomal region, and we raise the possibility of polymorphism in the genomic orientation of the BP1 to BP2 region. The identification of two new proteins *GOLGA8E* and *WHDC1L1* encoded by genes in the 15q11-q13 region may extend our understanding of the molecular basis of PWS. In terms of copy number variation and gene organization, this is one of the most polymorphic regions of the human genome, and perhaps the single most polymorphic region of this type.

## Background

Genomic alterations of the chromosome 15q11-q13 region are associated with two distinct genomic imprinting disorders, Prader-Willi syndrome (PWS) and Angelman syndrome (AS) [1]. PWS is characterized by neonatal hypotonia, childhood obesity, hypogonadism, moderate mental retardation, and behavioral problems. The most common molecular defect found in PWS patients is a ~6-Mb chromosomal deletion of the 15q11-q13 region on the paternal chromosome. Maternal uniparental disomy (UPD) of chromosome 15, microdeletions in a regulatory region known as the imprinting center (IC), and rare chromosome translocations have also been reported for PWS patients. It is clear from molecular studies that PWS is primarily caused by deficiency of a paternally expressed gene or genes from the 15q11-q13 region. However, it remains uncertain as to whether the major phenotypic features are caused by deficiency of one or more than one gene and whether such genes might be protein coding (e.g *NDN *or *MAGEL2*) or noncoding small nucleolar RNAs (snoRNAs) [2-4]. A 2-Mb region extending from the centromeric breakpoint 2 (BP2) to *D15S10 *was initially defined as the PWS candidate region [5]. Attempts to narrow the candidate region by characterizing several rare patients with cytogenetic abnormalities have been reported by several investigators [6-8]. However, a consensus concerning a narrowed critical region has not yet been reached. The controversy may arise from the complex regulation of a large imprinted domain as evidenced by IC mutations that have been reported to disrupt the imprinting process both in humans and in mice [9-17]. Alternatively, if PWS is a contiguous gene deletion syndrome, the individual genes may only contribute to part of the phenotype [18]. Numerous protein coding genes and non-coding transcripts have been isolated from the PWS candidate region. These include *SNURF-SNRPN, NDN, MKRN3, MAGEL2, PWRN1, PWRN2, IPW, PAR-1, PAR-4, PAR-5, C15orf2*, and multiple copies of different families of *snoRNA *genes [9,19-27]. All these transcripts except *PWRN2 *are expressed from the paternal chromosome with brain tissue-specific imprinting of *PWRN1 *and *C15orf2 *and therefore were considered as legitimate PWS candidate genes. Functional studies in mutant mice have suggested that *Ndn*, *Magel2*, or the *snoRNA *genes may play a role in the pathogenesis of PWS [2,28-30]. Lee et al. also determined the imprinting status of 22 transcripts located centromeric/proximal to the IC within the PWS candidate region based on an early version of the expressed sequence tag (EST) map and limited human genomic sequence. Seven of these transcripts were found to be paternally expressed but lacked protein coding potential [31]. Chai et al. identified four protein coding genes *CYFIP1, NIPA1, NIPA2*, and *GCP5 *in the proximal breakpoint region and established the genomic organization of the region between the two proximal breakpoints (BP1 and BP2) [32].

There have been multiple reports over the last decade describing a subset of fragile X syndrome patients who shared overlapping clinical features with PWS. These patients were often described as Prader-Willi-like fragile X syndrome [33-38]. The shared traits include extreme obesity, dysmorphic features, mental retardation and behavior problems. These patients have typical full mutations in the *FMR1 *gene indicating that the primary defect for the PWS-like phenotype was dysregulation of *FMR1*. The specificity of the PWS-like clinical features in fragile X syndrome patients has been debated [35].

Chromosome 15 is one of the seven human chromosomes with a high rate of segmental duplication [39]. Zody et al. carried out a detailed analysis of the duplication structure and history of chromosome 15 and reported that low copy repeats (LCRs), also termed segmental duplications (SDs), in chromosome 15 are largely clustered in proximal and distal 15q [40]. There are two breakpoints (BP1 and BP2) in the centromeric region and a single common breakpoint (BP3) in the telomeric region [41-46]. The remarkable consistency of the breakpoints strongly indicates the presence of a hot spot for recombination. Indeed, large genomic LCRs derived from the duplication of the actively transcribed *HERC2 *gene and its pseudogenes were identified in the BP2 and BP3 regions and are believed to contribute to a certain percentage of chromosomal rearrangements between 15q11 and 15q13 [18]. Recent reports suggest that *HERC2*-associated LCRs also are present within the BP1 region [32,47]. In addition, Pujana et al. have described a second cluster of LCRs (LCR15) with golgin-like protein (GLP) genomic sequence in the 15q11-q14, 15q24, and 15q26 regions [48,49]. Locke et al. also characterized the genomic structure and LCRs in the pericentromeric region of chromosome 15 proximal to BP1 [50].

Here we report an updated version of a sequence-based BAC contig covering the PWS candidate region based on the analysis of the finished version of the human genome sequence (NCBI Build 36.1), and we question whether the orientation of the BP1 to BP2 region may be polymorphic in the population. We provide further characterization of four protein coding genes, *CYFIP1*, *NIPA2, GOLGA8E, and WHDC1L1*, located at the proximal breakpoint of the PWS candidate region. *GOLGA8E *and *WHDC1L1 *are two protein coding genes from this region that are represented in genome databases but not previously discussed in the published literature. *CYFIP1 *and *NIPA2 *have been described [32] but we have undertaken further molecular characterization and reported new data regarding their gene structure and complex alternative splicing pattern. In addition, pulsed field gel electrophoresis (PFGE) analysis of the 15q11-q13 region has revealed a highly polymorphic region between BP1 and BP2 that is consistent with the presence of an abundance of copy number variants (CNVs) in the region as detected by genome-wide array CGH analysis [40,51].

## Results

### Sequence-based BAC contig covering the chromosome 15q11-q13 PWS candidate region

**Figure 1:**
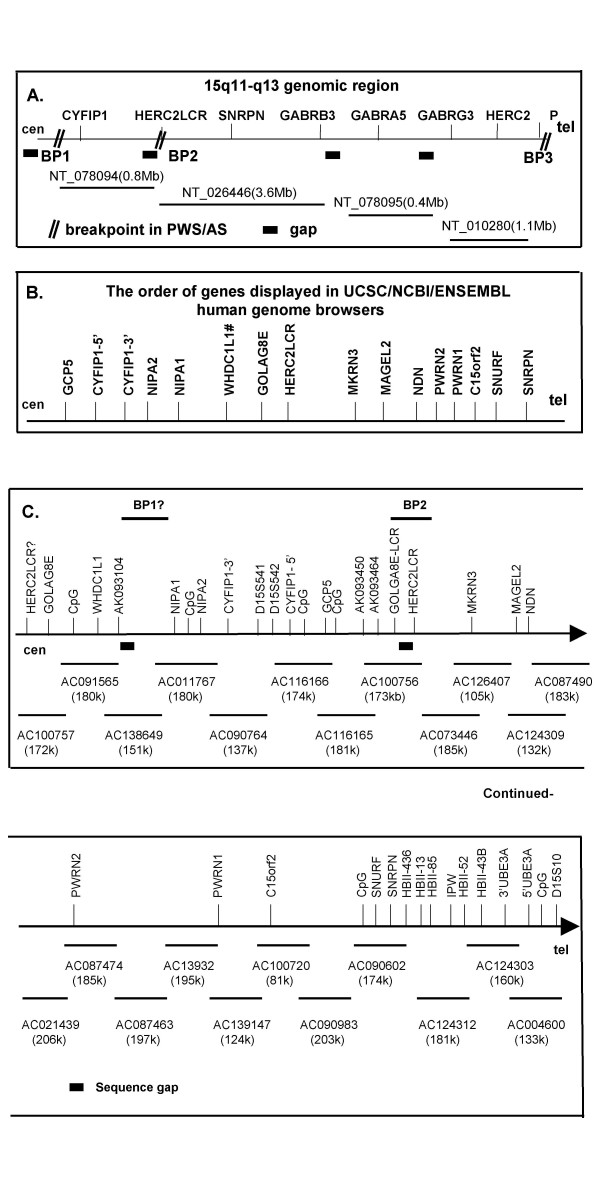
Sequenced BAC contig and transcriptional map of chromosome 15q11-q13. (A) Genomic super-contig. Four contigs are diagramed and four gaps are shown with no data shown centromeric to BP1. The exact sizes of the gaps are currently unknown. (B). The order of genes in the PWS candidate region displayed in UCSC, Ensembl, and NCBI human genome browsers. # indicates that *WHDC1LI *is only displayed in the UCSC browser but not in the Ensembl and NCBI browsers. (C) Detail of sequence-based BAC contig. Only one BAC representing each position is diagramed, and the scale is not proportional to genomic size. The size of each BAC was recorded from the current entry in Genbank and readers should refer to the database for the most recent information if necessary. Two sequence gaps are shown in the map as filled balck bars.

In an effort to annotate the 15q11-q13 region in detail and identify novel transcripts from the PWS candidate region, we first constructed a sequence-based BAC contig covering the PWS candidate region and then identified numerous overlapping ESTs and cDNA sequences primarily using bioinformatics tools. In the UCSC genome browser (NCBI Build 36.1) [52], there are four super genomic contigs covering the 15q11-q13 common deletion interval as diagramed in Fig. 1A (GenBank: NT_078094, NT_026446, and NT_010280). There are four sequence gaps between these contigs as diagramed in Fig. 1A. The exact size of these gaps is unknown but they are likely to be small based on the previously published data using YAC contigs [43]. The genomic size of 15q11-q13 deduced from the sequence contigs is around 6 Mb and is larger than the 4 Mb-extent frequently cited in the literature based on cytogenetic analysis and physical maps generated prior to the availability of the human genome sequence [5,43]. To construct a BAC contig covering the region, we first identified individual BAC clones using known genes and sequence tag site (STS) markers [44], as well as newly identified STS markers which were integrated into the UCSC genome browser; these were used as bait to search the NCBI High Throughput Genomic Sequence (HTGS) database [53]. The STS map reported by Christian et al. provided a scaffold for construction of the BAC contig [43]. A total of 65 minimal overlapping BAC clones were identified from a region between BP1 and the 5'-end of *UBE3A*, and the accession numbers of overlapping BAC clones are listed in Additional file 1. The BLAST 2 program [54] was then used to confirm the sequence overlap, and sequence identity >99.5% was considered to be significant. Although this limit was arbitrarily set, it was based on the estimation of the overall error rate of sequence from the human genome sequence project [55]. LCRs might have sequences that would fall within this degree of identity. In most regions, the contig shown in Fig. 1C is consistent with the genome assembly displayed in multiple databases and other reports [32,41,47]. However, the orientation of the BAC contig around BP1 is opposite to that of the contig displayed in the UCSC genome browser [52]. The exact orientation of this region remains uncertain and may even be polymorphic in the population. There are still two sequence gaps around the BP1 region as diagramed in Fig. 1 which illustrate the difficulties involved in both experimental manipulation and computational analysis due to the existence of highly identical LCRs within the region.

To further confirm the map position for the individual BAC clones assembled using bioinformatics tools, we performed FISH (fluorescence in situ hybridization) experiments using DNA derived from selected individual BAC clones from the region as probes. As listed in Additional file 1, the localization of 14 BAC clones to chromosome 15q was confirmed by FISH studies (data not shown). A simplified version of the BAC contig with only one BAC clone for each position is diagramed in Fig. 1C. The order of genes displayed in UCSC/Ensembl/NCBI human genome browsers is diagramed in Fig. 1B for reference and comparison [52,56,57]. A much more detailed description of the region including the BAC contig, STS markers, CpG islands, and the map positions of novel transcripts is summarized in Additional file 1.

### Characterization of four protein-coding genes from the proximal breakpoint of 15q11-q13 PWS candidate region

There are numerous ESTs mapped to this region, but most of them are intronless and/or lack major open reading frames (ORFs), and their significance cannot be easily addressed because they may function as noncoding RNAs or contain potential artifacts from cDNA production. In order to identify protein-coding genes, only transcripts with intron-exon structure and greater than 99% sequence identity between the EST and the matched genomic sequence were considered significant and are listed in Additional file 1. Towards this goal, we have analyzed each individual EST from this region for coding potential by ORF analysis. Most of the ESTs were overlapping and did not have significant coding potential, with the exception of clones [GenBank: BC063309, AK057421, BC018097, BC048987, D38549, BC011775, AK093450, AK093463, AK093104, AF272884, and BX537997] that displayed ORFs greater than 120 amino acids in length. There were no additional protein-coding genes identified in the region between *NDN *and *SNURF-SNRPN *despite the presence of numerous ESTs with clear intron-exon structure. In contrast, the region around the proximal breakpoints appears to have a high density of protein-coding genes based on our analysis using bioinformatics tools. To rule out the possibility of artifacts in these EST clones, we first performed expression analysis by reverse transcription polymerase chain reaction (RT-PCR) using RNA templates isolated from human lymphoblasts and brain tissue. Six representative cDNA clones [Genbank: D38549, BC011775, AF272884, BX537997, BC063309, and BC048987] were particularly interesting because of their clear protein-coding potential and readily detectable expression in lymphoblasts and brain tissues by RT-PCR. Clones D38549, AF272884, BC011775, and BX537997 were identical to four genes reported by Chai et al. which correspond to *CYFIP1*, *NIPA1*, *NIPA2*, and *GCP5*, respectively, while BC063309 and BC048987 are two protein-coding genes from this region that are predicted genes in the UCSC genome browser, but have not yet been characterized in the published literature. Computational analyses of EST clones AK093450, AK093104, and AK093463 predict significant ORFs, but we were not able to detect expression in either lymphoblasts or brain tissues by RT-PCR despite multiple attempts. We were also not able to detect any significant conservation when the sequences were compared to those available for multiple species, which raised a question concerning the functional significance of these EST transcripts. There are numerous additional ESTs within the region which show clear intron-exon structure and perfect sequence identity with the genomic sequence but do not contain significant ORFs, and their biological relevance remains to be defined. This analysis also confirmed the transcripts previously reported [31,58], but numerous additional ESTs without protein coding potential have since been deposited in the databases and mapped within the interval.

**Figure 2:**
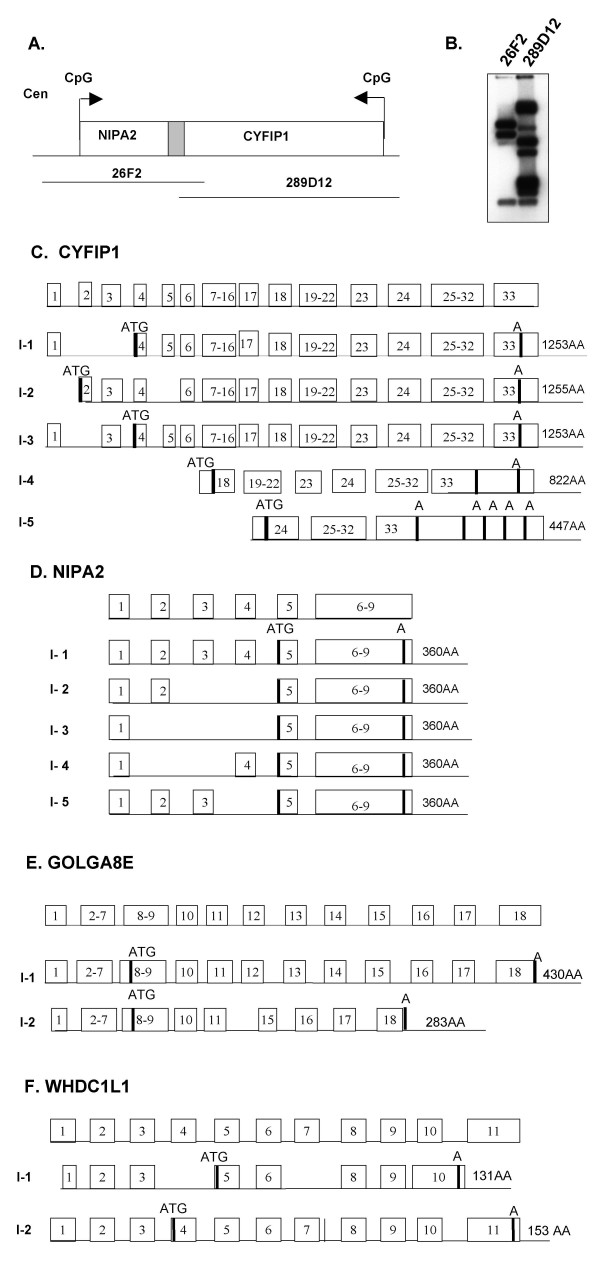
Genomic structure and alternative splicing of *CYFIP1*, *NIPA2, GOLGA8E*, and *WHDC1L1 *genes. (A) Physical map of *CYFIP1 *and *NIPA2 *genes. The two genes share a part of the 3' UTR region deduced from sequence analysis. (B) Confirmation of overlap between BAC 26F2 and 289D12 by Southern blot analysis. DNAs from BAC 26F2 and BAC 289D12 were digested with *Eco*RI and hybridized with a cDNA probe containing part of the *CYFIP1 *gene. (C) Alternative splicing and protein isoforms of *CYFIP1 *gene. (D) Alternative splicing and protein isoforms of *NIPA2 *gene. (E). Alternative splicing and protein isoforms of *GOLGA8E *gene. (F). Alternative splicing and protein isoforms of *WHDC1L1 *gene.

**Figure 3:**
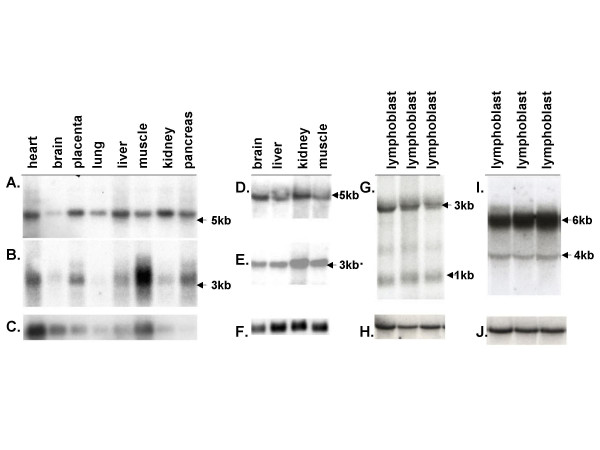
Northern blot analysis of gene expression for *CYFIP1*, *NIPA2, GOLGA8E*, and *WHDC1L1 *in human and mouse. (A and B) Hybridization of human *CYFIP1 (A) *and *NIPA2 *(B) probes to a Clonetech multiple human tissue northern blot with 2 ug poly (A+) RNA per lane. (D and E) Mouse northern blot analysis using total RNAs hybridized with *Cyfip1*(D) and *Nipa2*(E) probes. (G and I) Human northern blot analysis of RNA from cultured lymphoblasts collected for the Baylor human genome polymorphism project by using *WHDC1L1 *(G) and *GOLGA8E *(I) probes for hybridization. The results using a control glyceraldehyde-3-phosphate dehydrogenase (*GAPDH*) probe are shown in (C), (F), (H), and (J).

We have performed further analysis for two protein coding genes *CYFIP1 *and *NIPA2 *that have been previously reported by Chai et al. There were numerous EST and cDNA sequences identified by database searching using the cDNA clone D38549 encoding *CYFIP1 *that either overlapped, or increased the extent of the sequence at both the 5'- and 3'-ends and additional exons and an elongated 3'-untranslated region (UTR) were found. Alternatively spliced forms were identified by sequence alignment between the cDNA sequence and the genomic sequences. Further sequence analysis revealed five variant mRNA isoforms resulting from alternative 5'-splicing of the *CFYIP1 *gene that appeared to encode four different protein isoforms by ORF analysis (Fig. 2C). Isoforms 1 and 3 potentially encode the same protein with a length of 1253 amino acids. Isoform 2 has 1255 amino acids which is initiated from the most upstream ATG codon but skips exon 5. Isoforms 4 and 5 appear to be initiated from two different promoters within introns 17 and 23, respectively. These two isoforms also encode two shorter ORFs with a length of 822 amino acids for isoform 4 and 447 amino acids for isoform 5. The mRNA transcript for isoform 5 has the longest 3'-UTR containing five polyadenylation signals (AATAAA). It is not clear how the 5'-UTR is connected to the 3'-UTR for the different mRNA isoforms. Isoforms 4 and 5 appeared to have much longer 3'-UTRs when compared to isoforms 1, 2, and 3 based on the cDNA sequence currently deposited in the databases. Therefore, the total length of these cDNAs appeared to be similar to the other three isoforms despite lacking numerous 5'-exons. This may explain why there is a single signal from northern blot analysis as shown in Fig. 3A. The expression of isoforms 4 and 5 was confirmed by RT-PCR experiments using isoform-specific primers (data not shown). Interestingly, similar alternative splicing for isoform 4 is also found in mice [GenBank: BC086625] suggesting a functional significance for the alternative splicing. Sequence alignment between the cDNA sequence and genomic sequence indicated that *CYFIP1 *was comprised of 33 exons, and the pattern of alternative splicing and intron-exon junctions is diagramed in Fig. 2C and Additional file 2. *CYFIP1 *spans about 110 kb of genomic DNA. The cDNA sequence and protein isoforms were deposited in Genbank [AY763577–AY763580]. The majority of the 5'-exons mapped within BAC 289D12, and a small number of 3'-exons mapped to BAC 26F2. To examine the tissue-specific expression pattern of the *CYFIP1 *gene, northern blot analyses were performed using a 700-bp cDNA fragment generated by RT-PCR that corresponded to the 5'-end of the D39549 sequence. There was a strongly hybridizing 5.5-kb transcript seen in all tissues examined indicating that the cDNA sequence that we assembled most likely represented the full length of the *CYFIP1 *mRNA. The single band from northern analysis may represent many different transcripts with very similar size as revealed from computational analysis. *CYFIP1 *was expressed ubiquitously in numerous tissues examined (Fig. 3A). Similarly, we have also found five different splicing forms by *in silico *analysis that result from alternative splicing of the 5'-UTR of the *NIPA2 *gene but appear to encode the same protein based on ORF analysis. The variant *NIPA2 *mRNA sequences were also deposited in Genbank [AY732242] (Fig. 2D). An ORF with 360 amino acids was deduced from the available 2.8-kb cDNA sequence, and *NIPA2 *and *CYFIP1 *are transcribed towards each other but the transcriptional orientation to the centerome could not be determined definitely because these two genes are mapped within a very polymorphic region discussed in detail below. The intron-exon structures were determined and tabulated in Additional file 2. *NIPA2 *extends over about 30 kb of genomic DNA. The physical distance between the stop codons of *CYFIP1 *and *NIPA2 *is only 2.8 kb and interestingly, the 3'-UTR of *CYFIP1 *isoform 5 [GenBank: AY763579] and *NIPA2 *overlap (Fig. 2A).

For EST clone BC063309, a 5.3 kb-cDNA sequence was assembled after analysis of sequence homology with numerous overlapping EST clones. The expression of the transcript was confirmed by performing RT-PCR and sequencing the resulting product (data not shown). There are 18 exons based on the sequence alignment between the cDNA and genomic DNA and the intron-exon structure is summarized in Table 1. ORF analysis indicated that the cDNA BC063309 putatively encodes a protein 430 amino acids in length. The amino acid sequence is identical to the sequence of golgi autoantigen, golgin subfamily a, 8E (*GOLGA8E*) deposited in the database [Genbank: NM_001012423]. The *GOLGA8E *gene spans about 13 kb of genomic DNA and the transcriptional direction could not be determined definitely because of association with LCRs in the region. Expression of this gene is readily detectable in lymphoblasts and brain tissues by both RT-PCR (data not shown) and northern blot analysis with a predominant 6-kb transcript and a weaker 4-kb transcript as shown in Fig. 3I and 3J. Computational analysis also revealed two alternatively spliced forms for *GOLGA8E *as diagramed in Fig. 2E. Isoform 1 has an ORF of 430 amino acids and isoform 2 has an ORF of 283 amino acids. We have sequenced and confirmed part of the RT-PCR products and the annotated sequence for both isoforms has been deposited in the database. Protein database searching identified five other golgin subfamily genes within the 15q11-q14 and 15q24-q26 regions with identity of amino acid sequence ranging between 48–97% (see Table 2). However, protein searching for a conserved domain did not identify a coiled-coil domain that is believed to be characteristic for members of the golgin subfamily despite overall strong similarity to other golgin subfamily members.

**Table 1:** Intron-exon structure of *GOLAG8E*

**Exon**	**Exon (bp)**	**Intron (kb)**	**Intron**	**Exon**	**Exon**	**Intron**
1	146			AGTCGC	AGAAAG	tggctc
2	120	1.7	tggctc	TTAAAA	GGAGAT	tgacag
3	60	0.5	tgacag	TCAGCA	CTGGAG	ttgtag
4	81	0.2	ttgtag	AGCCCG	TCTTTG	ctcag
5	360	0.9	ctcag	AAACAA	CTAGAG	ccacag'
6	85	0.1	ccacag'	GTTCAA	TTGAAG	ccaaag
7	110	0.1	ccaaag	AAGAGT	GAGAGG	ttacag
8	90	1.1	ttacag	CAGTTC	ACACAG	ttgcag
9	108	0.1	ttgcag	TTGAAG	CAGGAG	gggcag
10	88	0.2	gggcag	GTTTGC	AGATGG	tctcag
11	257	0.4	tctcag	CTGAAC	GAGCTG	gtccag
12	69	0.3	gtccag	AACAAT	GGCAAG	ccttag
13	66	1.4	ccttag	GAGCGC	GGGAAG	caacag
14	92	0.4	caacag	GACACG	GCCATG	ctccag
15	101	0.6	ctccag	AGCGGC	CCATCA	ccgcag
16	101	0.1	ccgcag	GAAAGT	ATGAAG	ctccag
17	152	0.1	ctccag	GTGAAG	ATGGTG	ccaaag
18	1200	0.1	ccaaag	ATCTTT		

**Table 2:** The genome distribution of Golgin subfamily genes in human and mouse

Table	**Gene symbol**	accession No.	map position	genomic location	aa.	Iden%
**Human**	GOLGA8E*	NM_001012423	15q11	20986537–20999858	430	100
	GOLGA8A*	NM_015003	15q14	32458564–32487180	460	72
	GOLGA8B	NM_001023567	15q14	32604594–32613203	459	72
	GOLGA8G*	NM_01012420	15q13	26297495–26577158	260	97
	GOLGA	NM_018652	15q24.	70734092–70746791	693	49
	GOLGA6*	NM_010103840	15q26	72149251–72161944	693	48
	GOLGA1	NM_002077	9q33	126680450–126743178	767	22
	GOLGA2	NM_004486	9q34	130057930–130078089	430	47
	GOLGA3	AF485338	12q24	131859669–131908790	1498	22
	GOLGA4*	NM_002078	3p22	37259742–37383245	2230	25
	GOLGA5	NM_005113	14q32	92330403–92376057	731	23
	GOLGA7	NM_001002296	8p11	41467330–41487654	137	N
	GOLGB1	NM_004487	3q13	122865640–122951292	3259	24
	GORASP2	NM_15530	2q31	171493957–171531885	452	N
	GOLGA2L1	NM_017600	1223	99074326–99091202	144	36
	AF332229¶	AF332229	Yq11	24765503–24770366	184	45

**Mouse**	Golga1	NM_029793	2qB	126680478–126740968	758	20
	Golga3	NM_008146	59F	131859857–131908959	1447	25
	Golga4	NM_018748	9qF3	37259816–37383239	2238	24
	Golga5	NM_001033065	129E	92333536–92375541	729	25
	Golga7	NM_001042484	8qA2	41467233–41487467	137	N

* Indicates multiple isoforms for this protein but only one is shown in table, # the nucleotide position is referred to UCSC browser human genome build 36.1 and mouse genome build 36. aa, amino acid. Iden% of AA is the percentage of identity of amino acids for each gene deduced from BLAST 2 program when against to GOLGA8E. The 'N' indicates no significant sequence identity. ¶AF33229 is referred as Golgi autoantigen, golgin subfamily a2-like protein.

Using an approach similar to that described above, we also assembled a novel 3.2-kb cDNA sequence from cDNA clones [GenBank: BC048987/BC018097] and numerous overlapping EST sequences. The cDNA of BC048987 is located 140 kb distal to *GOLGA8E *and has multiple alternative mRNA forms. These different mRNA isoforms are associated with numerous ORFs, ranging in size from 42 amino acids to 153 amino acids based on computational analysis. The longest ORF has high similarity (72%) to a portion of the mouse *Whdc1 *protein (WAS protein homology region 2 domain containing 1; GenBank: NM_001004185) and thus was named *WHDC1L1 *(WAS protein homology region 2 domain containing 1-like 1). Comparison of the cDNA and genomic DNA sequences indicate that there are 11 exons, and two alternatively spliced forms with ORFs > 120 amino acids were identified and characterized in detail by sequence alignment between the cDNA and genomic sequences. We have confirmed the expression of this transcript in lympoblasts by RT-PCR (data not shown) and northern blot analysis (Fig. 3G and 3H). The intron-exon junctions and gene structure are summarized in Table 3 and alternative isoforms are diagramed in Fig. 2F. Isoform 1 has an ORF of 131 amino acids and isoform 2 has an ORF of 153 amino acids. We have resequenced some of the RT-PCR products and the annotated sequences for both isoforms were deposited in the database [Genbank: DQ309039 and DQ309040 for isoform 1 and isoform 2, respectively]. A protein database search revealed high identity (92%) to the middle part of hypothetical proteins (KIAA1971 protein, amino acids 333–446) encoded by two human cDNA clones [GenBank: AK126887/AB075851]. The KIAA1971 protein encoded by AK126887 is weakly similar to human JMY (junction-mediating and regulatory protein), a p300 cofactor involved in the regulation of the p53 response [59] and its mouse homolog named *Whdc1 *(WAS protein homology region 2 domain containing 1). Interestingly, AK126887 has been mapped to the 15q24 region while *Whdc1 *is located in a region homologous to human chromosome 15q24 in the central part of mouse chromosome 7. The possibility that the BC048987/BC018097 sequences found in the 15q11 region are part of a processed AK126887 pseudogene was ruled out by RT-PCR experiments using primers unique to the BC018097 sequence (data not shown).

**Table 3:** Intron-exon structure of *WHDC1L1*

**Exon**	**Exon (bp)**	**Intron (kb)**	**Intron**	**Exon**	**Exon**	**Intron**
1	656			CCGGAC	CTACAC	gtaagc
2	157	2.5	ctctag	GTTATT	CTTTAT	gtaagc
3	151	3.3	ctgtag	AAGTCC	TAGCAG	gtaata
4	159	0.7	tggcag	GAGTTT	AACTAG	gtgaga
5	170	0.2	taacag	GAATGC	GGAAAG	gtaaga
6	166	1.5	aataag	ATGGAA	TAAAAG	gtaaag
7	110	2.2	tttcag	ATGAAA	AACATG	gtatgt
8	187	1.3	ttttag	AAAAAC	AGAGAG	gtgggt
9	87	2.7	caccag	GATCAG	CAGATG	gtgagt
10	89	0.3	atatag	AAAAGA	AAAGAT	gtaagt
11	1694	2.5	ttaaag	AGAAAT	UTR	

### *GOLGA8E *associated LCR in 15q and other chromosomal regions

BLASTN searching of human genome sequence databases using the *GOLGA8E *cDNA as bait revealed numerous sequences with significant similarity across the 15q11-q14 and 15q24-q26 regions and sequence identities range from 91–97% at the nucleotide level as summarized in Additional file 3. There are also additional copies of sequence with sequence identity ranging from 50–89% that may represent different golgin subfamily genes in the 15q region (data not shown). BLAST searching also identified 6 BAC clones from chromosome 16 [GenBank: AC141597, AC141255, AC141254, AC141247, and AC140902], one BAC clone from chromosome 8 [GenBank: AC132916], and one BAC clone from chromosome 18 (AC136349) with sequence identity ranging from 90–97% compared to *GOLGA8E*. Careful sequence analysis revealed that the copy at genomic location chr15q11:20986537–20999858 bp (NCBI Build 36.1) proximal to the *CYFIP1 *and *NIPA2 *genes showed the highest identity to the genomic *GOLGA8E *DNA sequence providing evidence that the copy at this genomic position is actively transcribed. The rest of the copies distributed within the 15q11-q14, 15q24-q26, and other chromosomal regions are likely to be part of LCRs but the possibility of additional transcribed copies in other regions or an error in map position cannot be completely ruled out. There are five additional golgin subfamily genes mapped within the 15q13-q14 and 15q24-q26 regions. They are *GOLGA, GOLGA6, GOLGA8A*, *GOLGA8B*, and *GOLGA8G*. The map position and genomic location of these golgin subfamily genes in NCBI Build 36.1 are shown in Table 2. These results further support the initial characterization of LCR15 by Pujana et al. suggesting the presence of a new cluster of golgin like protein (GLP or GOLGA6) sequence-associated LCRs in this region [48,49]. We have provided further evidence that multiple actively transcribed golgin subfamily genes are associated with LCRs in the 15q region. The distribution of golgin subfamily genes associated with LCRs in the 15q11-q14 region suggests that these LCRs may contribute to the frequent long-range chromosomal rearrangements between the 15q11 region and the 15q24-q26 region reported in the literature [60].

### Mapping of *CYFIP1*, *NIPA2*, *GOLGA8E*, and *WHDC1L1 *to the proximal deletion breakpoint of the PWS/AS common deletion interval

**Figure 4:**
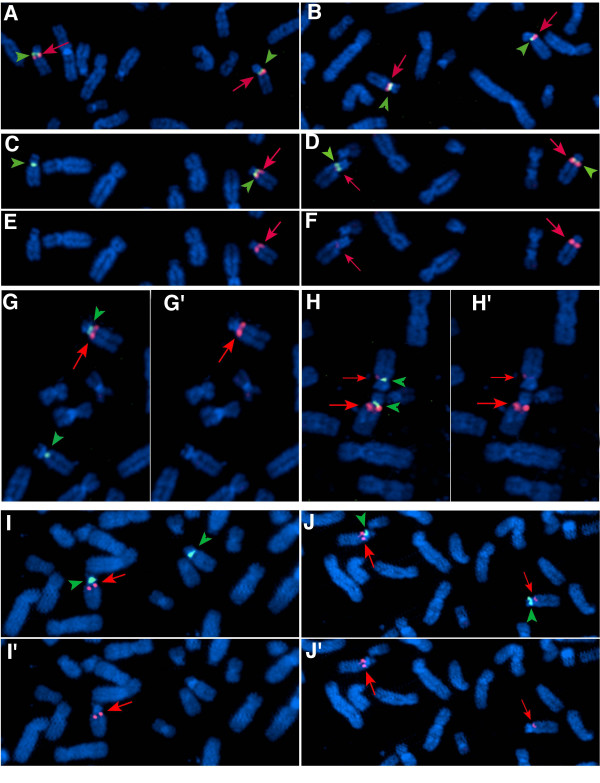
FISH analysis of PWS/AS patients with 15q11-q13 deletions. The chromosome 15-specific probe was labeled in green and BAC clones 26F2 and 289D12 were labeled in red. For (A), (B), (C), (D), (G), (H), (I,) and (J), the green and red signals were superimposed, while for (E), (F), (G'), (H'), (I'), and (J'), only the red signal was analyzed. BAC clone 289D12 was used as a probe for the images in the left column (A, C, E, G, G', I and I'), and BAC clone 26F2 was used as a probe for the images in the right column (B, D, F, H. H', J and J'). Three AS patients (AS-1, AS-2, and AS-3) and two PWS patients (PWS-1 and PWS-2) known to have large deletions of the 15q11-q13 region were analyzed; data for one PWS patient (PWS-1) is shown. Hybridization results for patient AS-1 are shown in (A) and (B), and for patient AS-2 in panels (C), (D), (E), and (F). Hybridization results for patient AS-3 are shown in (G), (G'), (H), and (H'), and panels (I), (I'), (J), and (J') are for patient PWS-1. For patient AS-1 in (A) and (B), signals for both BAC 26F2 and BAC 289D12 were seen on both chromosomes, indicating that the deletion is likely to be a class II deletion in this patient. For patient AS-2, the signals were absent for both BAC 26F2 and BAC 289D12 on one chromosome 15 as seen in (C) and (D), but a very dim red signal was seen for BAC 26F2 in one chromosome (F) when the red signal was analyzed separately which supports a partial deletion of 26F2 in these patients. Similar results were also seen for patients AS-3(G, G', H, and H') and PWS-1 (I, I', J and J').

Christian et al. reported that the order of several known STS markers relative to the centromere from microsatellite marker analysis was as follows: centromere-BP1-AC02B45-*D15S912*-*D15S18*-*D15S541*-*D15S542*-BP2-telomere [43,44]. It was suggested that the class I breakpoint mapped centromeric to *D15S541*/*D15S542 *and the class II deletion breakpoint was telomeric to *D15S541*/*D15S542 *based on the analysis of microsatellite markers in a large series of PWS/AS patients [44,45,61]. Sequence alignment between STS markers and the genomic sequence of BAC clones covering the region revealed that BAC 289D12 (AC90764) contained *D15S18*, *D15S541*, and *D15S542*, while *D15912 *and A002B245 mapped within BAC 26F2 (AC011767). In addition, *D15S541 *and *D15S542 *were found to map within *CYFIP1 *intron 1. The overlap between BAC 26F2 and BAC 289D12 revealed by sequence analysis was confirmed by Southern blot analysis using the insert of D38549 as a probe to hybridize to genomic DNA isolated from both BAC clones and digested with the restriction enzyme *Eco*RI (Fig. 2B). To determine whether *CYFIP1 *and *NIPA2 *were deleted in PWS/AS patients known to have large deletions of chromosome 15q11-q13, we performed FISH experiments on two PWS (PWS-1 and PWS-2) and three AS patients (AS-1, AS-2, & AS-3) using the BAC clones (289D12 and 26F2) where *CYFIP1 *and *NIPA2 *were identified (Fig. 4, data from one PWS patient (I, I', J& J') and three AS patients (A to H') are shown). These patients were known to have large chromosomal deletions of 15q11-q13 based on previous studies. Both BAC 26F2 and BAC 289D12 were detected as red signals on chromosome 15 when a centromere-specific probe from a commercial source was detected as a green signal. As shown in Fig. 4, only one red signal from the BAC 289D12 probe was detected in patient PWS-1 (I &I') and two AS patients (C & E for AS-2 and G & G' for AS-3) indicating a deletion of BAC 289D12 in these three patients who are likely to have a class I deletion. Two signals for the BAC 289D12 probe were present in patients AS-1 (A and B) and PWS-2 (data not shown) indicating that this BAC was not deleted. This is in agreement with a previous report that these patients have class II deletions [61]. For the BAC 26F2 probe, no signal or an extremely weak signal was visualized for patient PWS-1 (J & J') and two AS patients (AS-2 and AS-3) (D & H); all three patients were also deleted for BAC 289D12 by FISH. However, a very dim red signal from BAC 26F2 was apparent when images of red and green signals were analyzed separately (F & H'). This result suggests a possibility of partial deletion of 26F2 but the inherent limitations of FISH as a method for quantitation should also be considered. This result suggests that BAC 26F2 was likely to map centromeric to BAC 289D12 and BAC 26F2 was partially deleted in the AS/PWS patients analyzed. This observation is in agreement with the results reported by Pujana et al. using the same probes for FISH experiments, by others using array CGH, as well as the most recent data reported by Markoff and Flomen [41,47,48,62]. Because it was known from microsatellite analysis that these PWS/AS patients (PWS-1, AS-2, and AS-3) had class I deletions, we used the D38549 and *NIPA2 *inserts as probes to hybridize to DNA from these patients for Southern blot analysis. The dosage effect due to the deletion was revealed from genomic DNA Southern analysis but no abnormal junction fragments were detected (data not shown) indicating that the breakpoint is less likely to be within the *CYFIP1 *gene. The difficulty in identifying unique genomic probes from the breakpoint region precluded additional analysis such as multiple color interphase FISH to delineate the order of BAC clones within the BP1 region.

### Imprinting analysis for *CYFIP1 *and *NIPA2*

**Figure 5:**
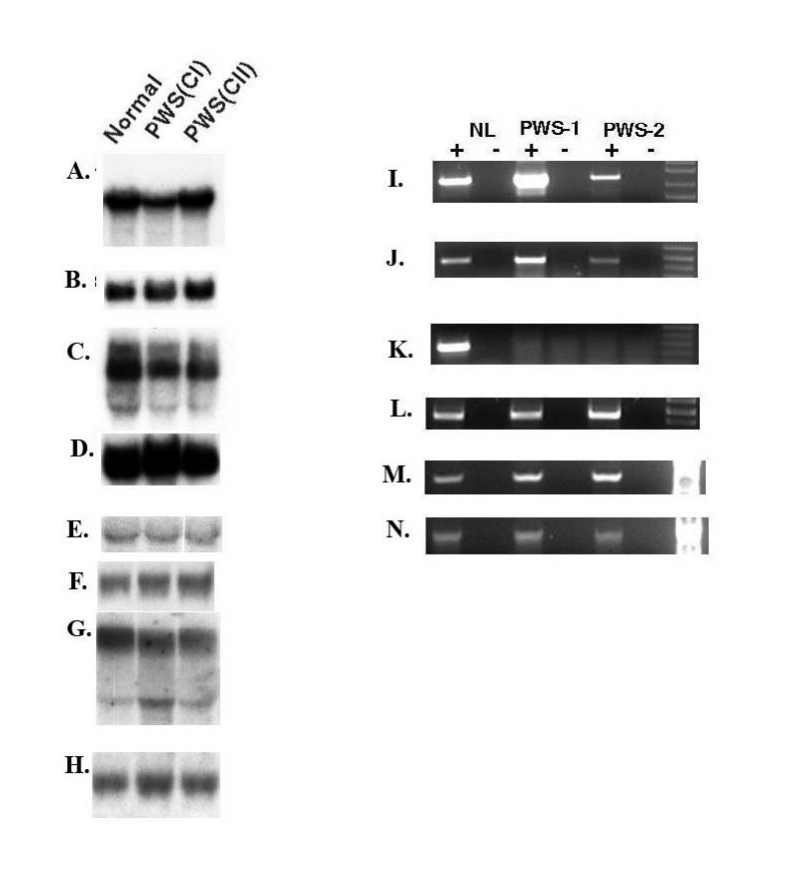
Imprinting analysis of *CYFIP1*, *NIPA2, GOLGA8E, and WHDC1L1*. Northern blot analysis of *CYFIP1 *and *NIPA2 *in PWS class I and class II deletion patients using cultured lymphoblasts. The expression of *CYFIP1*(A)*and NIPA2 *(C) was comparable in the normal control and class II (CII) patient but lower in the class I (CI) patient which is consistent with haplodeficiency of *CYFIPI *in class I patients suggesting these two genes are not subject to imprinting. The expression of *WHDC1L1 *in (E), and *GOLGA8E *in (G) is also comparable to the normal control but the interpretation for *WHDC1L1 *and *GOLGA8E *related to imprinting is complicated by the presence of many highly similar copies in the 15q region (see text for detailed discussion). The results using a control *GAPDH *probe are shown in (B), (D), (F), and (H). RT-PCR expression analysis of the brain tissues from PWS patients is shown for *CYFIP1 *in (I), for *NIPA2 *in (J), for *WHDC1L1 *in (M), and for *GOLGA8E *in (N). Both transcripts were detectable in both patients but appear to be lower in PWS-2. The absence of *SNRPN *expression in both PWS cases is confirmed in (K) and (L) is a *GADPH *control for RNA input. The "+" indicates a PCR reaction carried out with reverse transcriptase and "-" is without reverse transciptase. The molecular defect of PWS-2 is most consistent with maternal UPD of chromosome 15 or a rare possibility of an imprinting mutation because of the maternal methylation pattern at the *SNRPN *CpG island, the absence of expression of *SNRPN*, and the failure to detect a deletion by array CGH (data not shown). This data is also consistent with a report by others [66].

To examine whether imprinted expression is associated with *CYFIP1 *and *NIPA2*, RNA from cultured lymphoblasts derived from PWS patients with class I and class II deletions was used as a template for RT-PCR analysis. The expression of *GOLGA8E *and *WHDC1L1 *was also included in the analysis, but we were aware that it is probably impossible to determine the imprinting status for *GOLGA8E *and *WHDC1LI *in humans because of the presence of highly similar copies of these two genes within the region of 15q. The expression of *CYFIP1*, *NIPA2, GOLGA8E, and WHDC1L1 *was readily detectable in cultured lymphoblasts from PWS class I and II patients by RT-PCR analysis (data not shown). To further examine the possibility of quantitative or leaky imprinted expression for *CYFIP1*, *NIPA2*, *GOLGA8E*, and *WHDC1L1*, we also used total RNA derived from lymphoblasts from PWS patients with class I or class II deletions, and from normal control individuals for northern blot analysis. As shown in Fig. 5A and 5H, there was no significant difference in expression between patients with paternal deletions and normal controls for *CYFIP1, NIPA2*, *GOLGA8E, and WHDC1L1*. The decreased expression of *CYFIP1 *in class I deletion patients when compared to class II deletion patients is consistent with the conclusion that expression is not subject to imprinting. We then attempted to examine whether *CYFIP1, NIPA2, GOLGA8E, and WHDC1L1 *display a tissue-specific imprinting pattern particularly in brain as reported for other genes in the 15q11-q13 region including *UBE3A *and *ATP10A *[63-65]. We have obtained two brain tissues (PWS-1 and PWS-2) from the University of Maryland brain bank from individuals for whom a PWS diagnosis was initially made based on the clinical description. Molecular studies on these two cases were performed in the lab after we obtained the tissues. Both cases have abnormal DNA methylation at the *Not*I site within the *SNRPN *CpG island by genomic DNA Southern blot analysis (data not shown) which supports the clinical diagnosis of PWS [9]. In addition, array CGH analysis revealed a class II deletion in the case of PWS-1 but no apparent deletion of 15q11-q13 was detected in the case of PWS-2 which supports the possibility of maternal UPD of chromosome 15 or a rare imprinting mutation in the case of PWS-2 (T. Sahoo, personal communication). A similar conclusion was also reported by others [66]. We first examined the expression of *SNRPN *in these two patients as shown in Fig. 5K. The results of these experiments indicated that the *SNRPN *transcript was not detectable by RT-PCR which is consistent with the abnormal DNA methylation found at the *SNRPN *CpG island and again supports the diagnosis of PWS. We then examined the expression of *CYFIP1*, *NIPA2*, *GOLGA8E*, and *WHDC1L1 *in these two patients and transcripts from these four genes were detected by RT-PCR as shown in Fig. 5I, J, M, and 5N. The reduced intensity of the *CYFIP1 *PCR products in case PWS-2 may reflect the quality of the RNAs isolated from archival tissues. Analysis of gene expression by northern blot analysis in brain tissues was attempted but was not successful due to the poor quality of the RNA isolated from autopsy brain tissues. We have also examined the expression of *CYFIP1, NIPA2, GOLGA8E, and WHDC1L1 *using RNAs derived from lymphoblast cell lines from AS patients with a class I deletion and no evidence of allele-specific expression was found (data not shown). These data together with the results from deletion analysis support the conclusion that *CYFIP1 *and *NIPA2 *are not subject to imprinting in cultured lymphoblasts and brain tissues. These results are in agreement with those reported by Chai et al. [32] but a more complicated imprinting pattern such as cell type- or developmental stage-specific imprinting cannot be completely ruled out. Because of the presence of highly homologous sequences for *GOLGA8E *and *WHDC1L1 *in the 15q region, we were not able to determine whether *GOLGA8E *and *WHDC1L1 *have imprinted or biallelic expression.

### DNA methylation of CpG islands of *CYFIP1 *and *NIPA2*

Allele-specific differential methylation has been found consistently for multiple loci within the 15q11-q13 region where the paternal allele was usually unmethylated while the maternal allele was methylated. As described in the previous section, *CYFIP1 *and *NIPA2 *are localized within the common deletion interval described for PWS/AS patients but most likely lie at the most centromeric end of an imprinted domain within 15q11-q13. We have identified typical CpG islands in the 5'-region of the *CYFIP1*, *NIPA2, GOLGA8E*, and *WHDC1L1 *genes by computational analysis, and a detailed map of methylation-sensitive restriction enzyme sites was deduced (data not shown). To examine whether allele-specific differential DNA methylation was associated with the *CYF1P1 *CpG island, a DNA probe generated by PCR amplification of genomic DNA was used for Southern blot analysis after digestion with *Bss*HII in combination with *Hin*dIII. No allele-specific methylation was found after digestion of genomic DNA derived from any tissues examined, including several sub-regions of the brain and various cell lines, and both alleles appear to be completely unmethylated (data not shown). A similar result was also obtained for the *NIPA2 *CpG island where both alleles are also unmethylated (data not shown). However, we were not able to generate unique probes for genomic DNA Southern analysis for the *GOLGA8E *and *WHDC1L1 *CpG islands because of associated LCRs in the region.

### Pulse Field Gel Electrophoresis (PFGE) analysis of 15q11-q13 region

**Figure 6:**
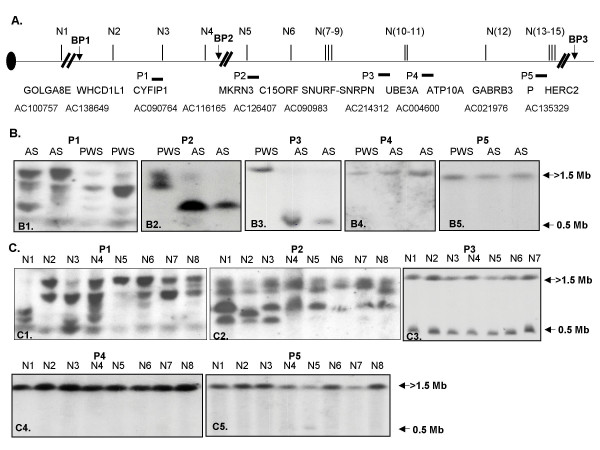
Polymorphic genomic region between BP1 and BP2 revealed by PFGE analysis. A. A *Not*I restriction enzyme map of the 15q11-q13 region revealed by computational analysis. There are total of 15 *Not*I sites identified and labeled as N1 to N15. N5 and N7–N9 were known to be differentially methylated with absence of methylation of the paternal allele and methylation of the maternal allele [9, 23, 68, 69] The known genes associated with *Not*I are diagramed below the line and the BAC clones where the *Not*I sites are mapped are shown. P1–P5 are genomic DNA probes used for PFGE analysis. B1-5. The result of PFGE analysis using *Not*I and genomic DNA probes P1–P5. The DNAs are from PWS and AS patients with large class I deletions. The pattern is consistent with allele-specific differential methylation of the *Not*I sites associated with *SNRPN *(N7-9) and *MKRN3 *(N5), methylation of both alleles for *C15orf2 *as previously reported, and the absence of methylation for the remainder of the *Not*I sites in the region. C1-5. The results of PFGE analysis using *Not*I and probes P1–P5 in normal individuals. The extensive variation revealed by probes P1 and P2 indicate a polymorphic genomic region between BP1 and BP2. The pattern of two bands shown in panel C3 using probe P3 is consistent with allele-specific differential DNA methylation of the *Not*I sites within the *SNRPN *CpG island (N7–N9). The lower band represents a genomic fragment (~500 kb) from the paternal chromosome between the CpG island of *SNRPN *and the CpG island of *UBE3A*.

The difficulty of generating unique probes for FISH analysis in the BP1 region prompted us to perform PFGE analysis to delineate the genomic structure for the BP1 region. A total of 15 *Not*I sites were identified between the BP1 and BP3 regions by computational analysis. The distribution of these *Not*I sites, the known genes associated with the *Not*I sites, and the BAC clones containing these *Not*I sites are diagramed in Fig. 6A. We then performed PFGE analysis using *Not*I and 5 different genomic probes generated from the 15q11-q13 region. The map position of 5 genomic probes is diagramed in Fig. 6A. These genomic probes except P2 were generated by PCR amplification from genomic DNAs and are located close to the CpG island of *CYFIP1 *(P1), the 3'-end of the *UBE3A *gene (P3), the CpG island of *ATP10A *(P4), and the CpG island of the *P *locus for albinism. The P2 probe is a previously reported cDNA clone named DN34 which represents part of the *MKRN3 *cDNA sequence (a gift from Dr. Daniel Driscoll). The *Not*I sites associated with the *SNURF*-*SNRPN *and *MKRN3 *CpG islands are known to be differentially methylated with the paternal allele unmethylated and the maternal allele methylated [9,24]. The *Not*I site associated with *C15orf 2 *(N6) was reported to be methylated on both alleles with a possibility of tissue-specific differences [23,27]. In contrast, the *Not*I sites associated with the *UBE3A *CpG island have been previously reported to be unmethylated for both alleles [67]. The results of the PFGE analysis using these probes are shown in Fig. 6B and 6C. The patterns revealed by using probes from the *MKRN3 *(P2) and 3'-*UBE3A *(P3) regions to analyze DNA derived from PWS and AS patients with large deletions are consistent with the results reported in the literature indicating differential methylation of *Not*I sites (N5 and N7–N9 in Fig. 6A) in these regions (Fig. 6B2–3) in which the paternal allele is unmethylated and the maternal allele is methylated [9,68,69]. The lower band shown in Fig. 6B3 represents the genomic fragment (~500 kb) from the paternal chromosome between the *SNRPN *CpG island and the *UBE3A *CpG island. The appearance of two closely migrating bands in the PWS sample when hybridized with the P2 probe suggests the presence of a genomic copy number variant proximal to *MKRN3 *and probably within the BP2 region (Fig. 6B2). A faint band in one of the AS samples may suggest a similar copy number variant or the possibility of incomplete restriction enzyme digestion. We have analyzed DNA from more than 20 anonymous normal individuals collected as part of the polymorphism project at Baylor, and representative data are shown in Fig. 6C1–5. There is no significant variation revealed using the P3, P4, and P5 probes in these individuals examined. However, extensive variation was revealed by using probes P1 and P2 among normal individuals. The pattern revealed by the P5 probe is somewhat surprising because of the known *HERC2*-associated LCR in the region. We were not able to discern the pattern and identity of each band by PFGE analysis because of the inability to generate a detailed physical map for the region due to the sequence gaps and the presence of the extensive LCRs in the region. Further analysis using different restriction enzymes and additional probes may help to delineate the genomic structure of the region but will still be challenging due to the presence of LCRs.

### CYFIP1, NIPA2, and GOLGA8E are members of different protein families

The functions of *CYFIP1*, *NIPA2*, *GOLGA8E*, and *WHDC1L1 *in mammals are unknown. To determine whether the CYFIP1 and NIPA2 proteins contain any functionally relevant or conserved protein motifs or domains, we carried out protein database searching (BLASTP) and protein motif and structural analysis (RPSBLAST) [70]. No conserved domain or motif was identified from this analysis. However, the protein structural analysis did reveal the presence of 5 and 8 transmembrane domains, respectively, for CYF1P1 and NIPA2. To examine the cross-species conservation for the CYFIP1 and NIPA2 proteins, the cDNA sequences and their predicted amino acid sequences were compared to multiple nucleotide and protein databases using the BLASTN and BLASTP programs [71]. Multiple significant sequence matches from other spcies including mouse, *Drosophila*, *C. elegans*, pig, *Fugu*, *Arabidopsis*, and *Anopheles gambiae *were identified and are diagramed in Additional file 4 for *CYFIP1 *and *NIPA2*, respectively. A human protein PIR121 was also identified that shows high similarity to CYFIP1 and was named CYFIP2 [72]. In addition, a variety of hypothetical proteins from *C. elegans*, *Drosophila*, and many other species were also identified for *CYFIP2*. *GOLGA8E *has significant similarity to many golgin subfamilies at the nucleotide and amino acid levels (Table 2). However, the protein structural analysis did not reveal a characteristic coiled-coil domain for GOLGA8E. There are total of 16 golgin subfamily genes found in the human genome and 6 of them are mapped to the proximal 15q region (Table 2). GOLGA8E has much higher similarity to the subfamily mapped in the 15q region than other subfamily genes in other chromosomal loci. In addition, there is no immediate evidence of evolutionary conservation in the mouse and other species for GOLGA8E despite the fact that several of its other family members appear to be highly conserved in mice (Table 2).

## Discussion

In an attempt to identify novel transcripts from the PWS candidate region, we have constructed a sequence-based BAC contig covering the ~2-Mb PWS candidate region. When compared to the previously published BAC/YAC contig covering the region of interest [32,41,44,47,73], we believe that this version of the BAC contig contributes significantly to the understanding of the physical map of the 15q11-q13 PWS candidate region. The current version of the map reported here is largely consistent with the maps reported by several other groups using the array CGH technique [32,41,42,47]. There are two sequence gaps remaining in the region around the proximal common PWS/AS deletion breakpoints (BP1 and BP2) in the sequence assembly available in the public domain. There are still three sequence gaps within the 6-Mb region, and closing these gaps has been challenging because of the extremely high sequence identity between different copies of LCRs. Makoff and Flomen recently reported closure of these gaps by an extensive bioinformatic approach [62]. The orientation of the contig drawn from this analysis differs from the contig displayed in the UCSC genome browser (NCBI Build 36.1) [52] in which BAC 289D12 was positioned centromeric to BAC 26F2 but is consistent with multiple reports [41,47,62]. The apparent discrepancy between the two genomic contigs may reflect an artifact introduced by using computational methodology because of the high sequence identity among the different LCRs or other aspects of the complex genome organization in the region. The structure depicted in the genome browsers is an attempt to select one unique order and copy number from data derived from two different chromosomes from multiple individuals, while the chromosomes may have a polymorphic gene order and copy number. This hypothesis is supported by a report that 9% of individuals in the general population have an inversion of the 15q11-q13 region [74], by the results from PFGE analysis reported in this study, as well as by the array CGH technique reported in the Database of Genomic Variants [75]. Our experience in utilizing the human genome sequence also emphasizes the importance of evaluating human genome sequence data in detail for a particular genomic region of interest before making any conclusion.

We have characterized transcripts for the first time for two protein-coding genes *GOLGA8E *and *WHDC1L1 *in addition to four other genes *CYFIP1, NIPA1, NIPA2*, and *GCP5 *previously described [32] from a region close to the proximal breakpoint (BP1) of the PWS candidate region. Our results for *CYFIP1 *and *NIPA2 *are largely in agreement with the previous report [32]. However, we have provided additional information regarding the genomic structure, alternative splicing, and expression pattern for both genes. These findings would be very important for understanding the function of CYFIP1 and its interaction with FMRP and Rac1 because isoforms 4 and 5 apparently lack the N-terminal FMRP and Rac1 interacting domain [76,77]. *GOLGA8E *and *WHDC1L1 *are associated with a large number of LCRs within the 15q11-q14 and 15q24-q26 regions. The function of *GOLGA8E *and *WHDC1L1 *is unknown. *GOLGA8E *belongs to the golgin subfamily which was originally identified as a group of Golgi-localized autoantigens recognized by sera from patients with a variety of autoimmune conditions [78]. There are l6 putative golgin subfamily genes in the human genome, but some of them have not yet been confirmed experimentally. These golgin subfamily members are differentiated mostly on the basis of molecular weight [78,79]. The function of most of golgin subfamily genes remains unclear, but a role for golgins in association with a GTPase protein complex in the organization and regulation of Golgi membrane trafficking has been suggested [80]. The presence of 6 out 16 golgin subfamily genes in the 15q11-q13 and 15q24-q26 regions is of interest relative to the possibility of actively transcribed genes with LCRs and the occurrence of LCR-mediated chromosomal rearrangements in the region. The golgin subfamily genes in 15q share higher similarity to GOLGA8E than other subfamily members on other chromosomes. Thus, golgin subfamily genes in 15q may have a common ancestry, and the SDs in the region may have played a role in the formation of new gene family members through evolution.

Although it is possible that *CYFIP1*and *NIPA2 *as well as *GOLGA8E *and *WHDC1L1 *may all be deleted in class I PWS patients, these four genes do not appear to be strong candidates for a primary role in the PWS phenotype. Our expression analysis using RNA isolated from the cultured lymphoblasts of PWS patients carrying class I deletions and brain tissues from patients with maternal UPD 15 suggest that *CYFIP1 *and *NIPA2 *are not subject to genomic imprinting. The possibility of cell type or developmental stage-specific imprinting cannot be completely ruled out but is less likely. In addition, a possibility that the paternal allele is more active was suggested by a recent study of the expression profile of *CYFIP1, NIPA1, NIPA2*, and *GCP5 *in class I and class II deletion patients using real time RT-PCR methods, but more study is needed to make a definitive conclusion [81]. It is impossible to analyze genomic imprinting for *GOLGA8E *and *WHDC1L1 *with high confidence because of multiple copies, some of which are distant from the BP1-BP2 region. Several recent reports comparing patients with class I and class II deletions have suggested that there are phenotypic differences between the two groups [81-83], while another investigator did not observe such distinctions [84]. Butler et al. recently found that PWS patients with class I deletions have a more severe phenotype than those with either a class II deletion or maternal UPD using a variety of psychological and behavioral tests [82]. Similarly, AS patients with class I deletions showed complete absence of vocalization while some AS patients with class II deletions were able to pronounce syllabic sounds [42,85]. These studies suggest that the deletion of biallelically expressed genes located between BP1 and BP2 such as *CYFIP1 *may have an impact on the degree of impairment particularly in communication and behavior observed for both PWS and AS patients.

Mapping of the *CYFIP1 *gene encoding an FMRP interacting protein within the PWS candidate region is of particular relevance for the analysis of the Prader-Willi-like phenotype observed in fragile X patients with full mutations in *FMR1*. The significance of the overlapping clinical features between PWS and fragile X syndrome remains uncertain, and it is very difficult to determine whether there is any intrinsic link underlying these overlapping clinical features solely from a clinical standpoint. Several recent studies report that autistic spectrum disorder is more prevalent in PWS patients than was previously thought [86-88]. The identification of *CYFIP1 *in the PWS candidate region may provide a molecular framework for further investigation, particularly in the area of shared autistic behavior between fragile X and Prader-Willi syndromes. Indeed, alteration of *CYFIP1 *in PWS-like fragile X syndrome patients was recently reported which provides a molecular basis for the clinical observation [89]. Altered expression of *CYFIP1 *has also been reported both in autism patients with maternal duplication of 15q11-q13 and in fragile X syndrome patients with autistic features [90].

Characterization of additional *GOLGA8E *LCRs around the BP1, BP3, and BP4 regions has added another layer of complexity in an already complicated region. The presence of an additional LCR (LCR15) other than *HERC2*-associated LCRs was first suggested by Pujana et al. [48] with the suggestion that golgin-like sequences are associated with LCR15. With the finished version of the human genome sequence, we were able to characterize the genomic organization and copy number of these LCRs in substantial detail. Like the *HERC2*-associated LCRs, this new class of LCR is associated with an actively transcribed copy of *GOLGA8E*. The *GOLGA8E*-associated LCRs have wider genomic distribution and are found in the 15q11, q13, q24, and q26, and other chromosomal regions while *HERC2*-associated LCRs are found in 15q11 and q13 and other chromosomes [46]. The finding of *GOLGA8E*-associated LCRs in the 15q11, q13, and q24-q26 regions suggests that these LCRs could be responsible for longer chromosomal rearrangements observed between the 15q11 and q24-q26 regions [60]. The degree of polymorphism in the BP1 and BP2 regions revealed by PFGE analysis is consistent with the data from multiple genome-wide analyses of CNVs by array CGH displayed in the Database of Genomic Variants or in other recent reports [40,47,50,74]. There are 14 different genomic variants listed in the region proximal to BP2 and most of these variants are SDs, but there are two inversion variants between BP1 and BP2, and one inversion variant between BP2 and BP3 was previously reported [74]. Murthy et al. recently reported a 250-kb heterozygous microdeletion covering the *CYFIP1 *and *NIPA1 *genes in the region between BP1 and BP2 in a male patient with mental retardation [91]. The interpretation of the finding is complicated because the same deletion was also present in the patient's father who may have had some mild phenotypic abnormalities. Butler et al. reported a PWS case with a typical class II deletion who also had a small duplication between BP1 and BP2, and the duplication was present in the healthy father and his brother [92]. We have observed that BAC 289D12 in the BP1 region detects a very frequent CNV in the cases referred for clinical array CGH analysis at Baylor College of Medicine (Cheung and Chaw, personal communication). In most of the cases, similar duplications were also found in one of the healthy parents and/or normal siblings. Clearly this is a highly polymorphic region. Very extensive investigations in normal individuals and in extended family members of patients with copy number gains or losses will be needed to determine if variations in this region sometimes cause abnormal phenotypes. Serious genotype/phenotype analysis for this region will require intensive copy number analysis at high resolution such as PFGE or fiber FISH to determine not only copy number but also orientation (regarding inversions), and perhaps even expression and epigenetic analysis.

## Conclusion

We have constructed and characterized a sequence-based BAC contig covering the PWS candidate region using human genome sequence databases. We have characterized transcripts for the first time for two protein-coding genes, *GOLGA8E *(golgin subfamily a, 8E) and *WHDC1L1 *(WAS protein homology region containing 1-like 1) and have further characterized two previously reported genes, *CYF1P1 *and *NIPA2*; all four genes are in the region close to the proximal/centromeric deletion breakpoint (BP1). GOLGA8E belongs to the golgin subfamily of coiled-coil domain proteins associated with the Golgi apparatus. *WHDC1L1 *is a novel gene with similarity to mouse *Whdc1 *(WAS protein homology region 2 domain containing 1) and human JMY protein (junction-mediating and regulatory protein). Biallelic expression of *CYFIP1 *and *NIPA2 *in cultured lymphoblasts and brain tissues analyzed suggests that they are unlikely to be major contributors to the pathogenesis of PWS, but haploinsufficiency may contribute to a more prominent behavioral phenotype seen in PWS and Angelman syndrome (AS) patients with class I deletions as compared to those with class II deletions. We have also characterized a new class of *GOLGA8E*-asssociated LCR in the 15q11-q13 and q24-q26 region. This class of LCR together with *HERC2*-assocaited LCRs may contribute to the frequent chromosomal rearrangements of 15q11-q13 and q24-q26 reported in the literature. This is one of the most polymorphic regions of the human genome in terms of copy number variation and gene organization, and perhaps the single most polymorphic region of this type.

## Methods

### RNA isolation

RNA was isolated from cultured human lymphocytes and human brain tissue, using Trizol reagent (GIBCO-BRL) according to the manufacturer's instructions.

### RT-PCR

For analysis of transcription, 3–5 ug total RNA treated with DNaseI was used for the reverse transcription reaction to synthesize single-stranded cDNA primed with random primers (GIBCO-BRL). The subsequent PCR reactions were performed using gene-specific primers as listed below.

### Northern analysis

Human northern blot analysis: A multiple human tissue blot was purchased from Clonetech (BD Bioscience) and hybridized with labeled probes following the manufacturer's recommendation. Mouse northern blot analysis: For each sample, 15 ug total RNA was pretreated with DNaseI and resolved on a 1.2% agarose gel in 10 mM NaPO_4 _buffer (pH 6.8) following glyoxal/DMSO denaturation using standard procedures. RNA was visualized by ethidium bromide staining and transferred to Hybond N+ membrane (Amersham Biosciences Corp.). Hybridization and washing conditions were the same as described below for genomic DNA Southern analysis.

### BAC DNA isolation, Genomic Southern blotting, and DNA methylation analysis

BAC DNA was isolated using a Qiagen Plasmid Maxi Kit (Qiagen Inc.) with some modifications of the original protocol. Five ug of BAC DNA was digested with *Eco*RI and separated on a 0.8% agarose gel prior to transfer to Hybond N+ membrane (Amersham Biosciences Corp.). For DNA methylation analysis by Southern blotting, genomic DNA was first isolated from lymphoblasts by phenol-chloroform extraction, and was then digested with *Sac*II and *Eco*RI in combination. The hybridization buffer was prepared as described [93], and the final washing condition for the membranes was a 20 minute rinse in a 0.2 × SSC and 1% SDS buffer.

### FISH

Metaphase chromosomes were prepared using standard protocols as described previously [94] and FISH was performed as previously described [95]. BAC DNA probes were labeled with digoxigenin and were detected by a standard protocol. DAPI counterstain was applied, and cells were viewed with a Zeiss Axiophot fluorescent microscope (Carl Zeiss Inc.) equipped with both single-band pass filters and a triple-band pass filter. Digital images were captured by a Power Macintosh G3 system and MacProbe version 4.0 or 4.3 (Perceptive Scientific Instruments). The chromosome 15 centromere Cep (satellite III) probe was labeled with spectrum green (Cytocell, Rainbow Scientific).

### PFGE analysis

High-molecular weight DNA was isolated and embedded in agarose plugs from peripheral blood samples and/or Epstein-Barr virus-transformed lymphoblastoid cells established from patients and their parents. DNA in plugs was digested by *Not*I (New England Biolabs, MA, USA). Separation of DNA fragments was achieved using a CHEF MAPPER (BioRAD) for 27 h with pulse time 86.54 s ramp at 6 V/cm. After treatment with 0.25 N HCl for 30 min and 0.4 N NaOH for 40 min, gels were blotted onto a nylon membrane. Radioactive probe labeling and hybridization were performed using the same protocol as described for genomic DNA Southern analysis.

### PCR Primer Sequences and Conditions

The sequences of the primers used for RT-PCR analysis of *CYFIP1 *were as follows: *CYFIP1*-2F: 5'-GTGCTGGATTTCTGCTACCATCTA-3' and *CYFIP1*-2R: 5'-GTCACAACTGGATTTAGTGGAAGC-3'. The cycling conditions were denaturation at 94°C for 10 min, followed by 35 cycles of 94°C for 45 sec, annealing at 56°C for 45 sec, and extension at 72°C for 45 sec with a final 5-min extension at 72°C. The PCR product was also used for northern analysis of *CYFIP1 *transcription. The sequences of the primers used for generating the probe for DNA methylation analysis of the *CYFIP1 *CpG island were as follows using the same cycling conditions as above: KICGF: 5'-ACAGACACCTGTCTTAACGCAGGA-3' and KICGR: 5'-TCTTGGAGAGAGGAGTTTTGGGCT-3'. The primers used for PFGE analysis were as follows. The P1 probe was generated using primers KICGF and KICGR. The P3 probe was generated by amplification with primers H3F: 5'-GACTTTCCACCTAACTCACTCAC-3' and H3R: 5'-GCAGCTGAGTGCCATATAATGTTG-3'. Probe P4 was generated using primers ATPCGF: 5'-AAGTAGTCTGTGGTCTGGCCCTTG-3' and ATPCGR: 5'-CAGACCTGGCTCAACTGGATAACG-3'. The P5 probe was generated by amplification with primers HP2F: 5'-TGAAATGCTCTTGCGTGGTTAGGA-3' and HP2R: 5'-AAATAAGGCATGCCCTCAGAGACA-3'. Analysis of *SNRPN *expression was carried out using the following oligonucleotides: *SNRPN *forward: 5'-CACCAGGCATTAGAGGTCCAC-3' and *SNRPN *reverse: 5'-GCAGAATGAGGGAACAAAAACCT-3'. The cycling conditions were denaturation at 94°C for 1 min, annealing at 63°C for 1 min., and extension at 72°C for 1 min [9].

The PCR primers used for amplification of *WHDC1L1 *were as follows: forward: 5'-TCG GAA GTG AAA GAA CTC AGA AGG-3'; reverse: 5'-AGA CTG AGG ATC ATT TTG TGG AGG-3'. The PCR conditions were denaturation at 94°C for 45 sec, annealing at 63°C for 45 sec., and extension at 72°C for 1 min.

The sequences of the PCR primers used for the analysis of *GOLGA8E *were as follows: forward: 5'-AGC GTA CTA CAG TTG GAG CAG CAA-3'; reverse: 5'-ATC TCC TTC TTC TTG GCA GCC AAG-3'. The PCR conditions were denaturation at 94°C for 45 sec, annealing at 63°C for 45 sec., and extension at 72°C for 45 sec.

## Authors' contributions

YJ carried out the design, sequence analysis, the molecular studies, and preparation of the manuscript. KW, CK, and LS carried out the FISH studies. QL and HL carried out molecular studies of *CYFIP1 *and *NIPA1*. YP carried out the molecular studies of *GOLGA8E *and *WHDIL1*. AB and JB participated in the design, supervision, and preparation of the manuscript. All authors read and approved the final manuscript.

## Supplementary Material

Additional file 1 **Table S1**. Overlapping BAC clone, STS makers, ESTs cross the PWS candidate region Click here for file

Additional file 2 **Table S2A**. Intron-exon structure of *CYFIP1 *gene. **Table S2B**. Intron-exon structure of *NIPA2 *gene Click here for file

Additional file 3 **Table S3**. The distribution of *GOLGA8E *associated low copy repeats in the15q11-q14 and 15q24-q26 regions Click here for file

Additional file 4 **FigS1**. Cross-species conservation of *CYFIP1 *(A) and *NIPA2 *(B) gene families. Click here for file
