# Supplementary material for: Genomic analysis of the chromosome 15q11-q13 Prader-Willi syndrome region and characterization of transcripts for GOLGA8E and WHCD1L1 from the proximal breakpoint region
Source: BMC Genomics. 2008 Jan 28;9:50. doi: 10.1186/1471-2164-9-50 (PMC2268926; doi:10.1186/1471-2164-9-50)
Supplement: Additional file 1 — Table S1. Overlapping BAC clone, STS makers, ESTs cross the PWS candidate region [file 1471-2164-9-50-S1.ppt]

## Slide 1
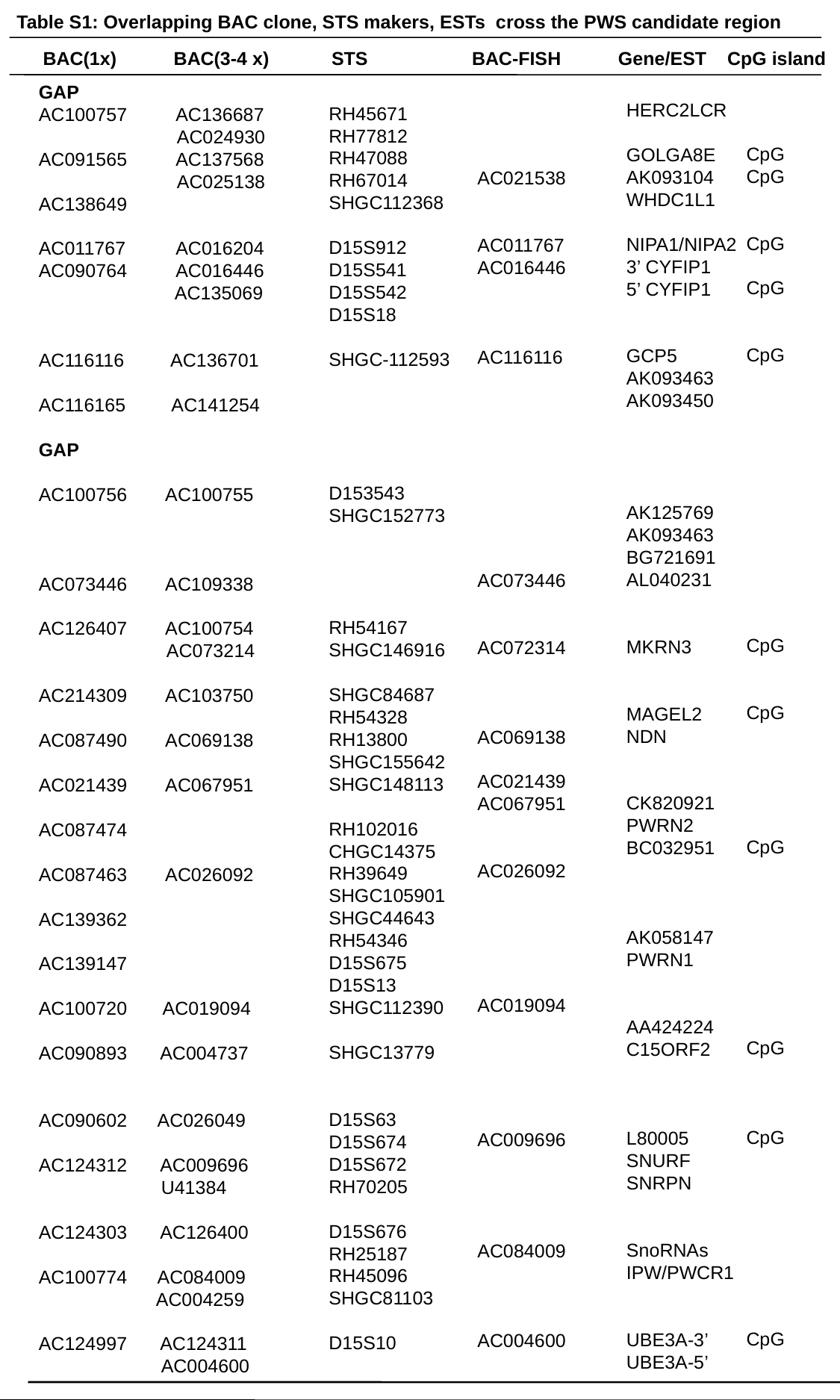

Table S1: Overlapping BAC clone, STS makers, ESTs cross the PWS candidate region
 BAC(1x) BAC(3-4 x) STS BAC-FISH Gene/EST CpG island
CpG
CpG
CpG
CpG
CpG
CpG
CpG
CpG
CpG
CpG
CpG
HERC2LCR
GOLGA8E
AK093104
WHDC1L1
NIPA1/NIPA2
3’ CYFIP1
5’ CYFIP1
GCP5
AK093463
AK093450
AK125769
AK093463
BG721691
AL040231
MKRN3
MAGEL2
NDN
CK820921
PWRN2
BC032951
AK058147
PWRN1
AA424224
C15ORF2
L80005
SNURF
SNRPN
SnoRNAs
IPW/PWCR1
UBE3A-3’
UBE3A-5’
AC021538
AC011767
AC016446
AC116116
AC073446
AC072314
AC069138
AC021439
AC067951
AC026092
AC019094
AC009696
AC084009
AC004600
RH45671
RH77812
RH47088
RH67014
SHGC112368
D15S912
D15S541
D15S542
D15S18
SHGC-112593
D153543
SHGC152773
RH54167
SHGC146916
SHGC84687
RH54328
RH13800
SHGC155642
SHGC148113
RH102016
CHGC14375
RH39649
SHGC105901
SHGC44643
RH54346
D15S675
D15S13
SHGC112390
SHGC13779
D15S63
D15S674
D15S672
RH70205
D15S676
RH25187
RH45096
SHGC81103
D15S10
GAP
AC100757	 AC136687
	 AC024930
AC091565	 AC137568
	 AC025138
AC138649
AC011767	 AC016204
AC090764	 AC016446
 AC135069
AC116116	 AC136701
AC116165 AC141254
GAP
AC100756	 AC100755
AC073446	 AC109338
AC126407	 AC100754
	 AC073214
AC214309	 AC103750
AC087490	 AC069138
AC021439	 AC067951
AC087474
AC087463	 AC026092
AC139362
AC139147
AC100720 AC019094
AC090893	 AC004737
AC090602 AC026049
AC124312	 AC009696
	 U41384
AC124303	 AC126400
AC100774 AC084009
	 AC004259
AC124997	 AC124311
	 AC004600
