# Supplementary material for: Genomic analysis of the chromosome 15q11-q13 Prader-Willi syndrome region and characterization of transcripts for GOLGA8E and WHCD1L1 from the proximal breakpoint region
Source: BMC Genomics. 2008 Jan 28;9:50. doi: 10.1186/1471-2164-9-50 (PMC2268926; doi:10.1186/1471-2164-9-50)
Supplement: Additional file 2 — Table S2A. Intron-exon structure of CYFIP1 gene. Table S2B. Intron-exon structure of NIPA2 gene [file 1471-2164-9-50-S2.ppt]

## Slide 1
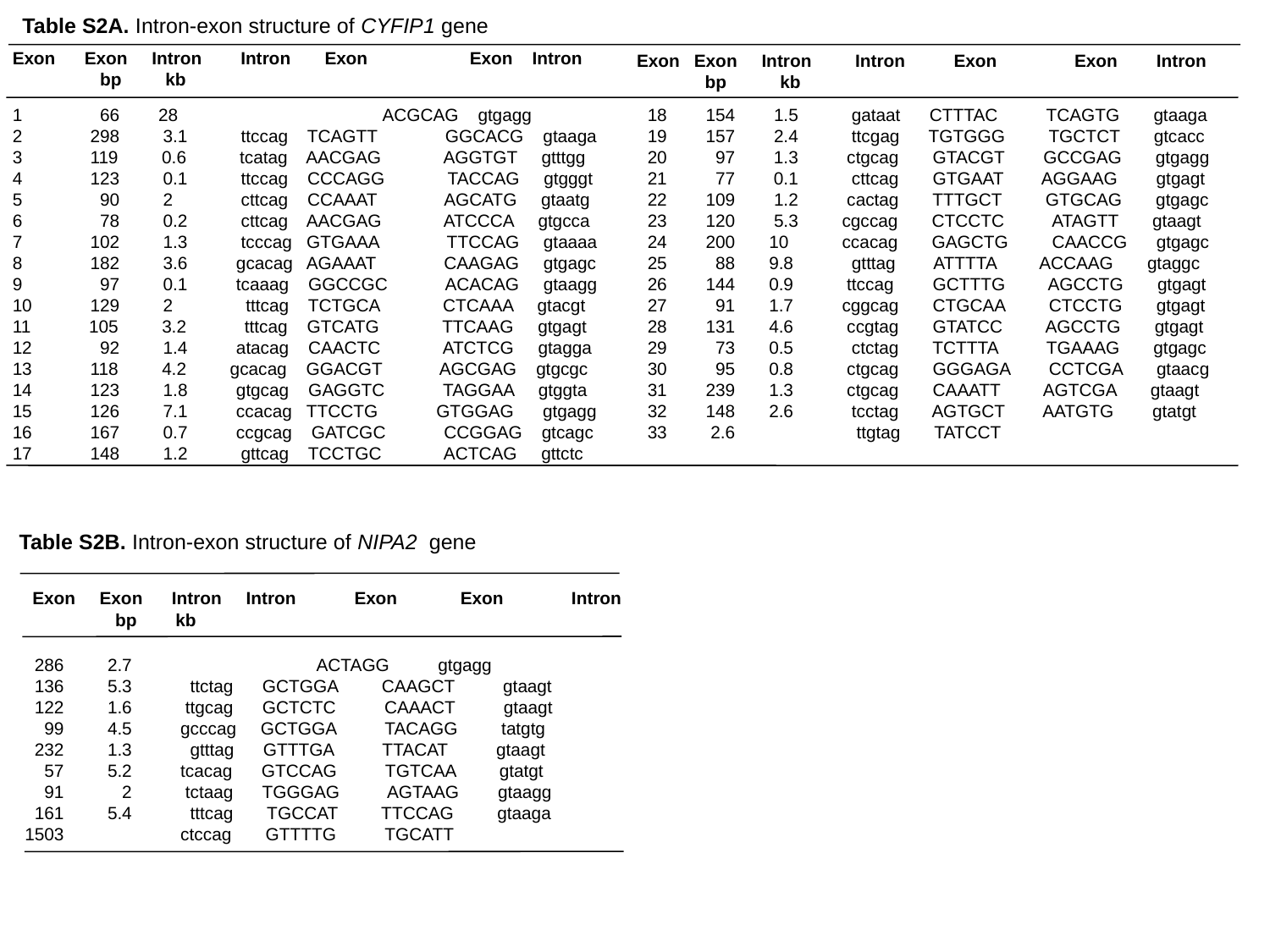

Table S2A. Intron-exon structure of CYFIP1 gene
Exon Exon Intron Intron Exon Exon Intron
 bp kb
Exon Exon Intron Intron Exon Exon Intron
 bp kb
1 66 28 		 ACGCAG gtgagg
2 298 3.1 ttccag TCAGTT GGCACG gtaaga
3 119 0.6 tcatag AACGAG AGGTGT gtttgg
4 123 0.1 ttccag CCCAGG TACCAG gtgggt
5 90 2 cttcag CCAAAT AGCATG gtaatg
6 78 0.2 cttcag AACGAG ATCCCA gtgcca
7 102 1.3 tcccag GTGAAA TTCCAG gtaaaa
8 182 3.6 gcacag AGAAAT CAAGAG gtgagc
9 97 0.1 tcaaag GGCCGC ACACAG gtaagg
10 129 2 tttcag TCTGCA CTCAAA gtacgt
11 105 3.2 tttcag GTCATG TTCAAG gtgagt
12 92 1.4 atacag CAACTC ATCTCG gtagga
13 118 4.2 gcacag GGACGT AGCGAG gtgcgc
14 123 1.8 gtgcag GAGGTC TAGGAA gtggta
15 126 7.1 ccacag TTCCTG GTGGAG gtgagg
16 167 0.7 ccgcag GATCGC CCGGAG gtcagc
17 148 1.2 gttcag TCCTGC ACTCAG gttctc
18 154 1.5 gataat CTTTAC TCAGTG gtaaga
19 157 2.4 ttcgag TGTGGG TGCTCT gtcacc
20 97 1.3 ctgcag GTACGT GCCGAG gtgagg
21 77 0.1 cttcag GTGAAT AGGAAG gtgagt
22 109 1.2 cactag TTTGCT GTGCAG gtgagc
23 120 5.3 cgccag CTCCTC ATAGTT gtaagt
24 200 10 ccacag GAGCTG CAACCG gtgagc
25 88 9.8 gtttag ATTTTA ACCAAG gtaggc
26 144 0.9 ttccag GCTTTG AGCCTG gtgagt
27 91 1.7 cggcag CTGCAA CTCCTG gtgagt
28 131 4.6 ccgtag GTATCC AGCCTG gtgagt
29 73 0.5 ctctag TCTTTA TGAAAG gtgagc
30 95 0.8 ctgcag GGGAGA CCTCGA gtaacg
31 239 1.3 ctgcag CAAATT AGTCGA gtaagt
32 148 2.6 tcctag AGTGCT AATGTG gtatgt
33 2.6 ttgtag TATCCT
Table S2B. Intron-exon structure of NIPA2 gene
Exon Exon Intron Intron Exon Exon Intron
 bp kb
1 286 2.7 	 ACTAGG gtgagg
2 136 5.3 ttctag GCTGGA CAAGCT gtaagt
3 122 1.6 ttgcag GCTCTC CAAACT gtaagt
4 99 4.5 gcccag GCTGGA TACAGG tatgtg
5 232 1.3 gtttag GTTTGA TTACAT gtaagt
6 57 5.2 tcacag GTCCAG TGTCAA gtatgt
7 91 2 tctaag TGGGAG AGTAAG gtaagg
8 161 5.4 tttcag TGCCAT TTCCAG gtaaga
9 1503 ctccag GTTTTG TGCATT
