# Supplementary material for: Genomic analysis of the chromosome 15q11-q13 Prader-Willi syndrome region and characterization of transcripts for GOLGA8E and WHCD1L1 from the proximal breakpoint region
Source: BMC Genomics. 2008 Jan 28;9:50. doi: 10.1186/1471-2164-9-50 (PMC2268926; doi:10.1186/1471-2164-9-50)
Supplement: Additional file 3 — Table S3. The distribution of GOLGA8E associated low copy repeats in the15q11-q14 and 15q24-q26 regions [file 1471-2164-9-50-S3.ppt]

## Slide 1
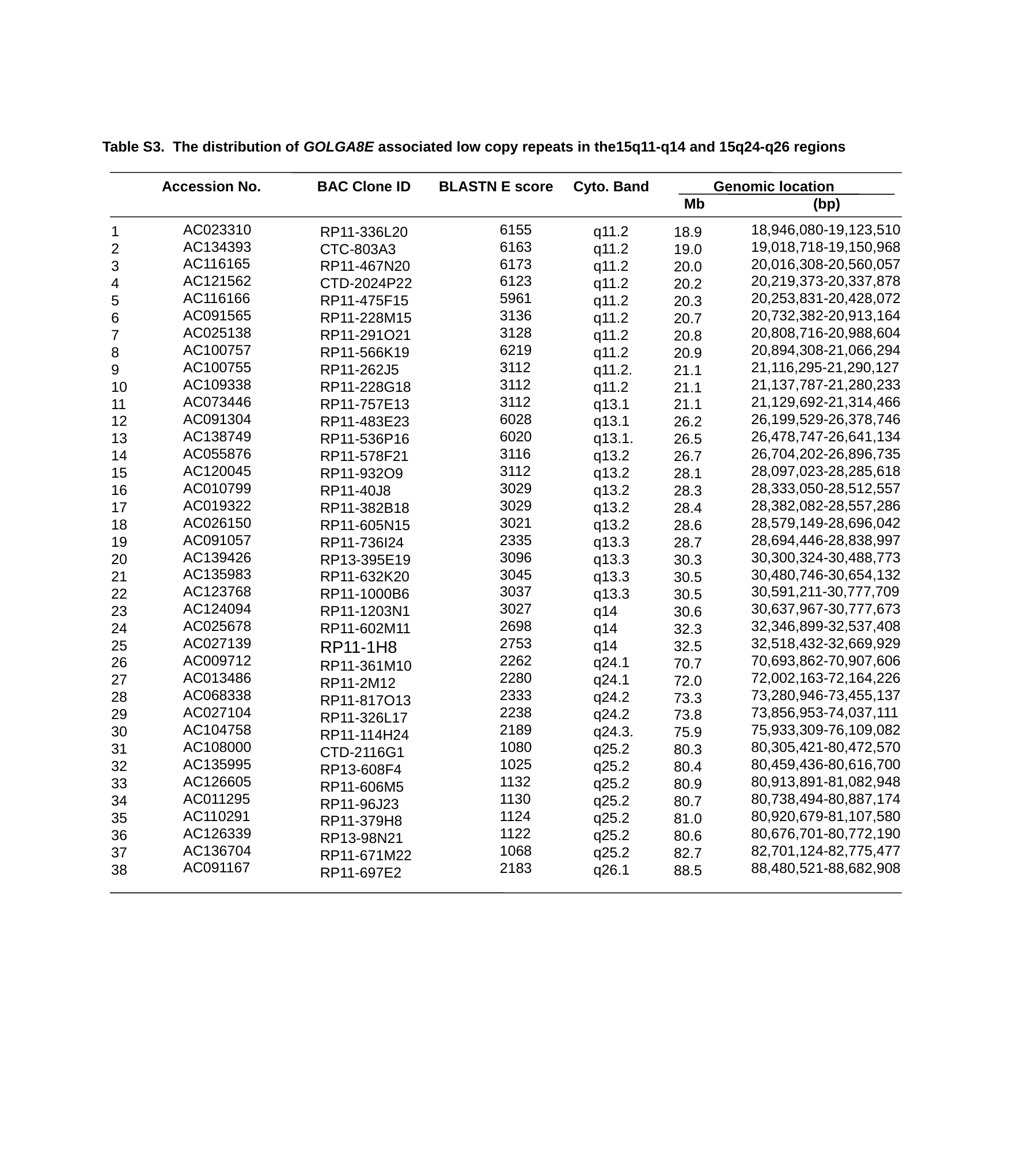

Table S3. The distribution of GOLGA8E associated low copy repeats in the15q11-q14 and 15q24-q26 regions
 Accession No. BAC Clone ID BLASTN E score Cyto. Band Genomic location
						 Mb (bp)
AC023310
AC134393
AC116165
AC121562
AC116166
AC091565
AC025138
AC100757
AC100755
AC109338
AC073446
AC091304
AC138749
AC055876
AC120045
AC010799
AC019322
AC026150
AC091057
AC139426
AC135983
AC123768
AC124094
AC025678
AC027139
AC009712
AC013486
AC068338
AC027104
AC104758
AC108000
AC135995
AC126605
AC011295
AC110291
AC126339
AC136704
AC091167
6155
6163
6173
6123
5961
3136
3128
6219
3112
3112
3112
6028
6020
3116
3112
3029
3029
3021
2335
3096
3045
3037
3027
2698
2753
2262
2280
2333
2238
2189
1080
1025
1132
1130
1124
1122
1068
2183
18,946,080-19,123,510
19,018,718-19,150,968
20,016,308-20,560,057
20,219,373-20,337,878
20,253,831-20,428,072
20,732,382-20,913,164
20,808,716-20,988,604
20,894,308-21,066,294
21,116,295-21,290,127
21,137,787-21,280,233
21,129,692-21,314,466
26,199,529-26,378,746
26,478,747-26,641,134
26,704,202-26,896,735
28,097,023-28,285,618
28,333,050-28,512,557
28,382,082-28,557,286
28,579,149-28,696,042
28,694,446-28,838,997
30,300,324-30,488,773
30,480,746-30,654,132
30,591,211-30,777,709
30,637,967-30,777,673
32,346,899-32,537,408
32,518,432-32,669,929
70,693,862-70,907,606
72,002,163-72,164,226
73,280,946-73,455,137
73,856,953-74,037,111
75,933,309-76,109,082
80,305,421-80,472,570
80,459,436-80,616,700
80,913,891-81,082,948
80,738,494-80,887,174
80,920,679-81,107,580
80,676,701-80,772,190
82,701,124-82,775,477
88,480,521-88,682,908
1
2
3
4
5
6
7
8
9
10
11
12
13
14
15
16
17
18
19
20
21
22
23
24
25
26
27
28
29
30
31
32
33
34
35
36
37
38
q11.2
q11.2
q11.2
q11.2
q11.2
q11.2
q11.2
q11.2
q11.2.
q11.2
q13.1
q13.1
q13.1.
q13.2
q13.2
q13.2
q13.2
q13.2
q13.3
q13.3
q13.3
q13.3
q14
q14
q14
q24.1
q24.1
q24.2
q24.2
q24.3.
q25.2
q25.2
q25.2
q25.2
q25.2
q25.2
q25.2
q26.1
RP11-336L20
CTC-803A3
RP11-467N20
CTD-2024P22
RP11-475F15
RP11-228M15
RP11-291O21
RP11-566K19
RP11-262J5
RP11-228G18
RP11-757E13
RP11-483E23
RP11-536P16
RP11-578F21
RP11-932O9
RP11-40J8
RP11-382B18
RP11-605N15
RP11-736I24
RP13-395E19
RP11-632K20
RP11-1000B6
RP11-1203N1
RP11-602M11
RP11-1H8
RP11-361M10
RP11-2M12
RP11-817O13
RP11-326L17
RP11-114H24
CTD-2116G1
RP13-608F4
RP11-606M5
RP11-96J23
RP11-379H8
RP13-98N21
RP11-671M22
RP11-697E2
18.9
19.0
20.0
20.2
20.3
20.7
20.8
20.9
21.1
21.1
21.1
26.2
26.5
26.7
28.1
28.3
28.4
28.6
28.7
30.3
30.5
30.5
30.6
32.3
32.5
70.7
72.0
73.3
73.8
75.9
80.3
80.4
80.9
80.7
81.0
80.6
82.7
88.5
