# Supplementary material for: Genomic analysis of the chromosome 15q11-q13 Prader-Willi syndrome region and characterization of transcripts for GOLGA8E and WHCD1L1 from the proximal breakpoint region
Source: BMC Genomics. 2008 Jan 28;9:50. doi: 10.1186/1471-2164-9-50 (PMC2268926; doi:10.1186/1471-2164-9-50)
Supplement: Additional file 4 — FigS1. Cross-species conservation of CYFIP1 (A) and NIPA2 (B) gene families. [file 1471-2164-9-50-S4.ppt]

## Slide 1
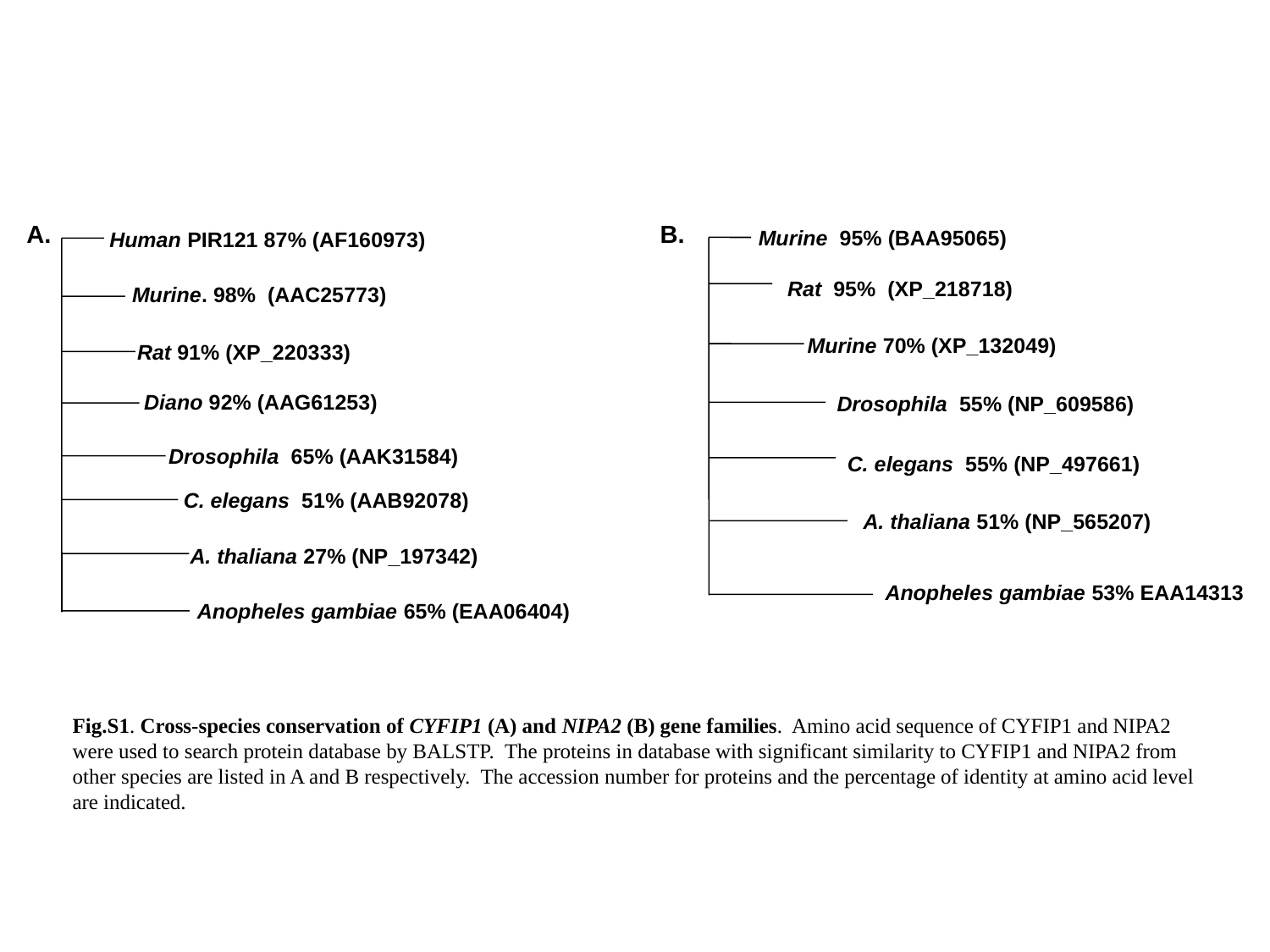

A.
B.
Murine 95% (BAA95065)
Human PIR121 87% (AF160973)
Rat 95% (XP_218718)
 Murine. 98% (AAC25773)
Murine 70% (XP_132049)
Rat 91% (XP_220333)
Diano 92% (AAG61253)
Drosophila 55% (NP_609586)
Drosophila 65% (AAK31584)
C. elegans 55% (NP_497661)
C. elegans 51% (AAB92078)
A. thaliana 51% (NP_565207)
A. thaliana 27% (NP_197342)
Anopheles gambiae 53% EAA14313
Anopheles gambiae 65% (EAA06404)
Fig.S1. Cross-species conservation of CYFIP1 (A) and NIPA2 (B) gene families. Amino acid sequence of CYFIP1 and NIPA2 were used to search protein database by BALSTP. The proteins in database with significant similarity to CYFIP1 and NIPA2 from other species are listed in A and B respectively. The accession number for proteins and the percentage of identity at amino acid level are indicated.
